# Bacterial DNA is present in the fetal intestine and overlaps with that in the placenta in mice

**DOI:** 10.1371/journal.pone.0197439

**Published:** 2018-05-17

**Authors:** Keith A. Martinez, Joann Romano-Keeler, Joseph P. Zackular, Daniel J. Moore, Robert M. Brucker, Christopher Hooper, Shufang Meng, Naoko Brown, Simon Mallal, Jeff Reese, David M. Aronoff, Hakdong Shin, Maria Gloria Dominguez-Bello, Jörn-Hendrik Weitkamp

**Affiliations:** 1 Department of Microbiology, Sackler Institute, New York, New York, United States of America; 2 Department of Translational Medicine, NYU School of Medicine, New York, New York, United States of America; 3 Department of Pediatrics, Vanderbilt University Medical Center, Nashville, Tennessee, United States of America; 4 Department of Pathology, Microbiology & Immunology, Vanderbilt University Medical Center, Nashville, Tennessee, United States of America; 5 Department of Biological Sciences, Vanderbilt University, Nashville, Tennessee, United States of America; 6 Division of Infectious Diseases Department of Medicine, Vanderbilt University Medical Center, Nashville, Tennessee, United States of America; 7 Institute for Immunology and Infectious Diseases, Murdoch University, Murdoch, Western Australia; 8 Department of Cell and Developmental Biology, Vanderbilt University, Nashville, Tennessee, United States of America; BC Children's Hospital, CANADA

## Abstract

Bacterial DNA has been reported in the placenta and amniotic fluid by several independent groups of investigators. However, it's taxonomic overlap with fetal and maternal bacterial DNA in different sites has been poorly characterized. Here, we determined the presence of bacterial DNA in the intestines and placentas of fetal mice at gestational day 17 (n = 13). These were compared to newborn intestines (n = 15), maternal sites (mouth, n = 6; vagina, n = 6; colon, n = 7; feces, n = 8), and negative controls to rule out contamination. The V4 region of the bacterial 16S rRNA gene indicated a pattern of bacterial DNA in fetal intestine similar to placenta but with higher phylogenetic diversity than placenta or newborn intestine. Firmicutes were the most frequently assignable phylum. SourceTracker analysis suggested the placenta as the most commonly identifiable origin for fetal bacterial DNA, but also over 75% of fetal gut genera overlapped with maternal oral and vaginal taxa but not with maternal or newborn feces. These data provide evidence for the presence of bacterial DNA in the mouse fetus.

## Introduction

While bacterial colonization of the *in utero* environment is well recognized during preterm labor and preterm rupture of membranes [1–4] a long-standing dogma establishes a sterile *in utero* environment during undisturbed term healthy pregnancy [5]. However, recent studies incorporating culture independent techniques have found bacterial DNA in the placenta, amniotic fluid, and meconium [2,6–13]. In addition, we discovered higher bacterial DNA diversity in the presumably sterile small intestinal tissue samples surgically resected shortly after birth for congenital intestinal obstruction compared to fecal samples from the same infants [14]. Shared features between bacterial DNA in placenta and amniotic fluid with infant meconium and the influence of maternal diet on the newborn microbiome suggest microbial transfer at the feto-maternal interface [10,15]. However, presence of bacterial DNA in amniotic fluid, placenta and postnatal meconium is not direct evidence for its existence in the fetal intestine. Therefore, we aimed to detect bacterial DNA in the fetal gut and to study its likely origin. While bacterial DNA does not infer viable bacteria, its presence may be critical for the developing immune system.

Because contaminating DNA has been detected in DNA extraction kits and other laboratory reagents, caution is required in studies of low biomass samples, such as the feto-maternal unit [16]. Here we carefully controlled for exogenous contamination, and provide community membership estimates, their overlap at multiple maternal-fetal body sites and attempted to source-track these taxonomic members using computational methods.

## Results

### Fetal intestines harbor bacterial DNA with rich diversity

**Fig 1:**
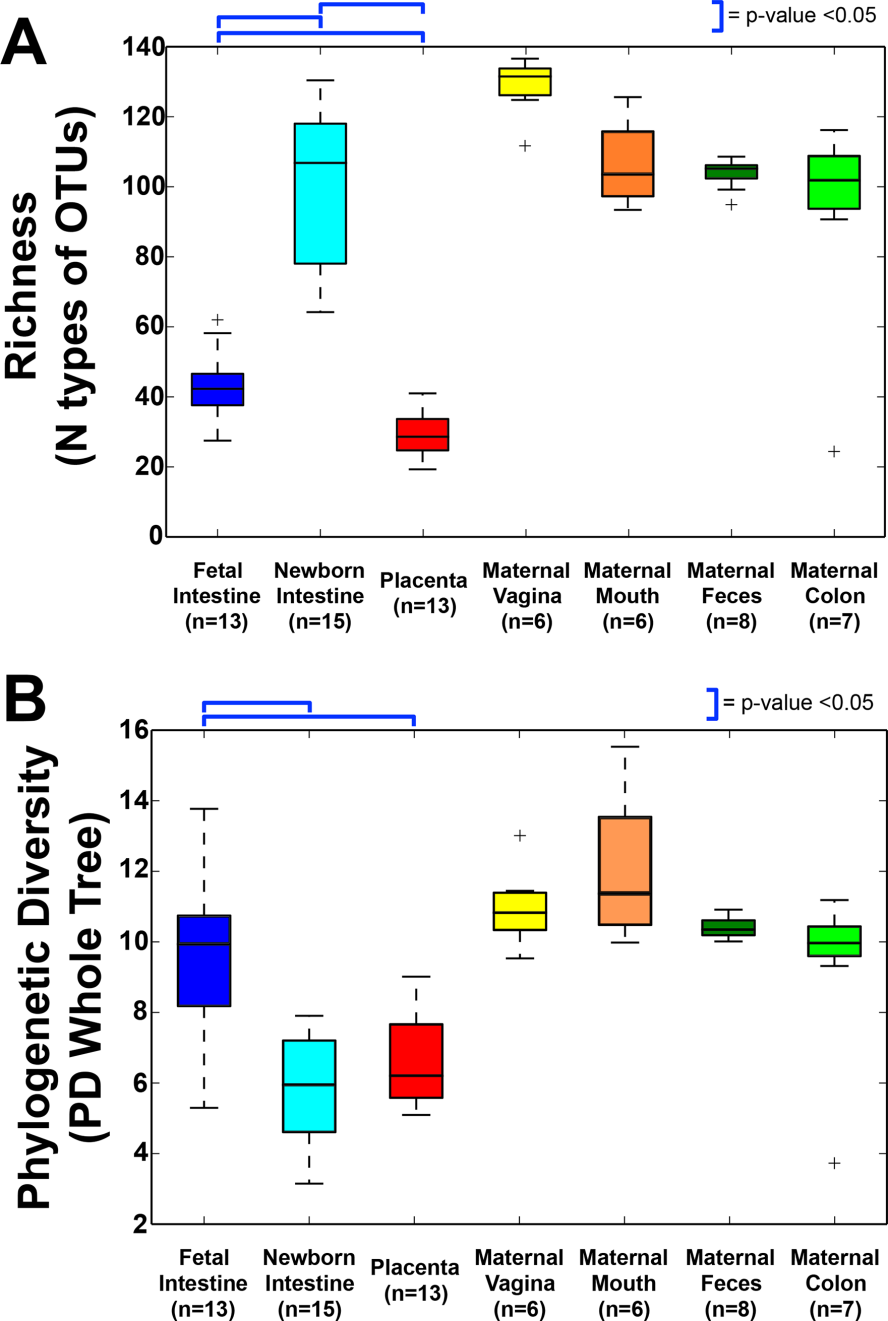
Alpha diversity of bacterial DNA from fetal, newborn and maternal samples. (A) Richness (observed species metric) (B) Phylogenetic diversity, accounting for richness, evenness, and phylogeny. Figure generated in QIIME. Blue brackets indicate comparisons with p-values <0.05, paired sample t-test.

In this study, we used a mouse model of normal pregnancy to determine the presence of 16S rRNA gene sequences in fetal and newborn intestines, and in placental and maternal (oral, vaginal, colon, feces) samples. The number of samples analyzed for each body site is shown in Fig 1. We collected maternal, placental, fetal samples from 4 individual dams and their pregnancy products after sacrifice for sterile C-section and maternal and neonatal samples from 4 individual dams and their pups delivered by vaginal birth. Dams/litters were housed individually.

We used real time PCR to validate 16S rRNA load across samples with presumed low bacterial biomass. As reflected by cycle threshold (C_t_), the amount of relative bacterial biomass detected in the fetal intestines at gestational day 17 (E17) was higher than placental samples at the same time point (C_t_ = 25.95 versus mean C_t_ = 28.63). Assuming perfect PCR efficiency, these differences equate to eight-fold higher bacterial DNA content in the fetal intestine compared to placenta. As expected, the greatest relative bacterial biomass was detected in maternal stool samples on E17 (mean C_t_ = 20.73) and postnatal day 1 (P1, mean C_t_ = 19.84).

After subtracting the OTUs found in blank samples and negative controls, we found bacterial DNA in fetal and newborn intestines, placenta, and all maternal samples (oral, vaginal, feces). PERMANOVA analysis showed a statistical difference between the bacterial DNA found in negative controls when compared to samples from fetal intestines and placentas (p-value < 0.001, data not shown). Bacterial DNA in the fetal intestine showed higher richness (100 ± 47 OTUs) than in the placenta (75 ± 31; p-value < 0.05), and newborn intestine (75 ± 38, p-value < 0.05; Table 1). Maternal sites (vaginal and fecal) had the highest richness (Fig 1A) and phylogenetic diversity (Fig 1B).

**Table 1:** Sequencing depth and OTU counts for maternal, fetal, placental and newborn samples.

Sample Type	Maternal				Fetal		Newborn	Controls		
	Feces	Colon	Oral	Vagina	Placenta	Intestine	Intestine	PCR Control	DNA Ext. Control	Total
**N samples**	8	7	6	6	13	13	15	2	7	136
**Total N sequences to pick OTUs**	47,271	9,151	46,625	35,662	21,480	23,482	5,764	6,811	557,518	1,116,193
**Mean N of sequences ±SD**	5,909 ±2,409	1,307 ±675	7,771 ±1,107	5,944 ±3,703	1,652 ±953	1,806 ±1,031	384±208	3,406 ±4,523	79,645 ±60,626	
**N of OTUs represented**	3,074	1,009	5,344	2,915	863	1,172	379	326	4,094	17,484
**Mean N OTUs ±SD**	789 ±178	238 ±83	1,688 ±255	714 ±415	75 ±31	100 ±47	75 ±38	171 ±91	816 ±509	

**Fig 2:**
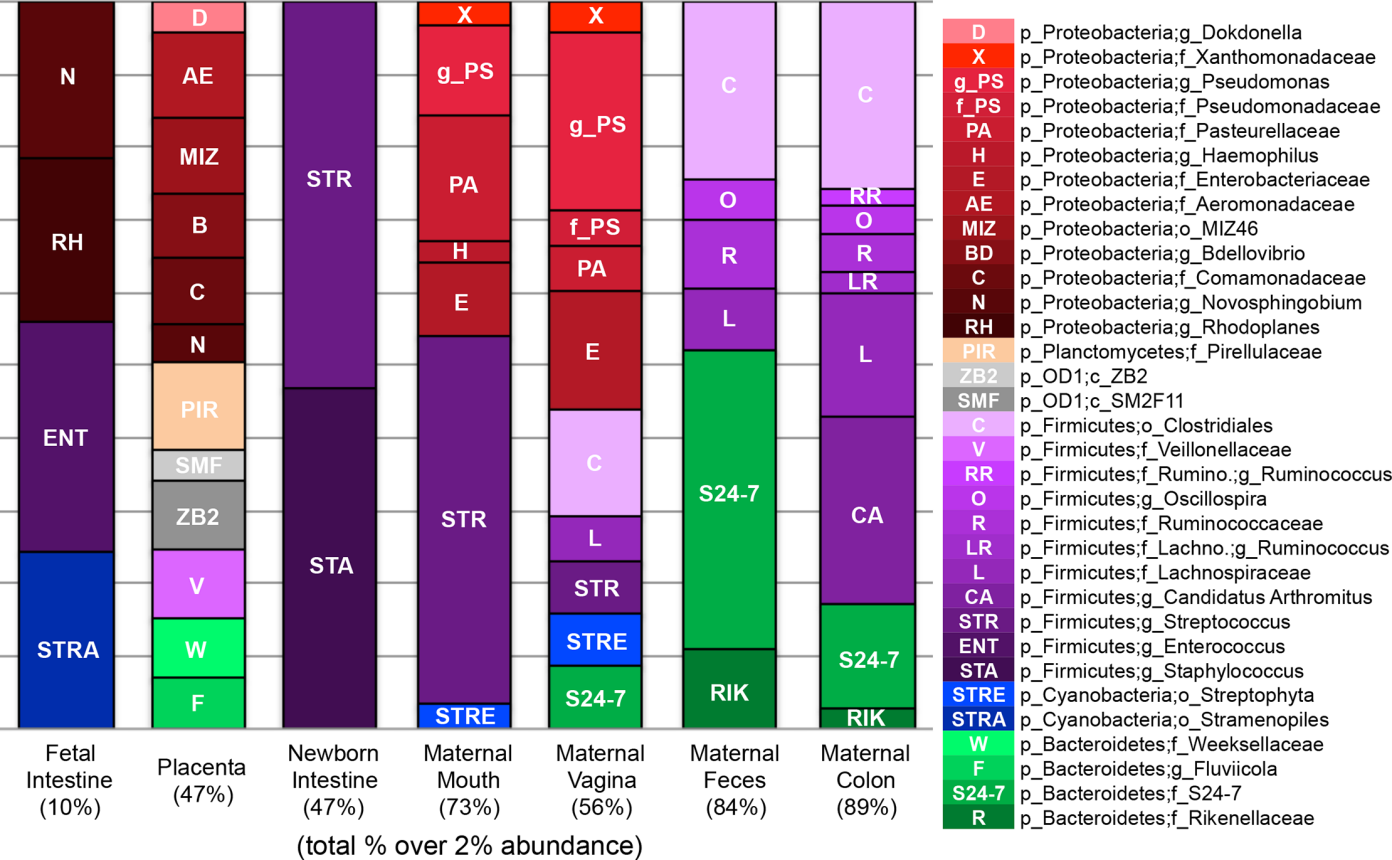
Major bacterial taxa to which fetal, newborn and maternal bacterial DNA were assigned. Taxa present at ≥ 2% relative abundance. Taxonomies are the lowest level of identification provided by OTU classification. Figure generated in QIIME.

Fetal intestines had higher proportions of *Enterococcus* (3.2%) and lower proportions of *Streptococcus* and *Staphylococcus* in relation to the newborn intestine, maternal mouth or vagina (Fig 2). The bacterial DNA composition in the fetal intestine was highly even, with 90% of taxa represented at <2% relative abundance (Table 2). Conversely, most taxa found in the maternal mouth and vagina were found at >2% relative abundance (73% and 56%, respectively). Interestingly, 22% of the genera found in the fetal intestine were unique to that site, while only 14% were unique to the placenta. Phylotype analysis using Ribosomal Database Project classification (RDP) [17] confirmed the results of Greengenes analyses (S1 Fig). The most abundant fetal intestine bacterial signature was from *Lachnospiraceae*, a common gut bacterium in mammals. In addition, fetal intestines and placenta had the greatest abundance of *Burkholderiaceae* DNA. Postnatally, the intestine became dominated by *Streptococcus* and *Staphylococcus* (S2 Fig).

**Table 2:** Percentage of taxa with relative abundance in each percentile by sample type.

Sample Type	Percent Taxa with Relative Abundance in each Percentile		
	Major Taxa ≥ 2%	Minor Taxa 2%–0.1%	Sub-taxa ≤ 0.1%
Fetal Intestine	10	47	43
Placenta	47	46	7
Newborn Intestine	47	13	40
Maternal Mouth	73	20	7
Maternal Vagina	56	37	7
Maternal Feces	84	15	1
Maternal Colon	89	9	2

### The majority of fetal intestinal bacterial DNA was of placental origin

**Fig 3:**
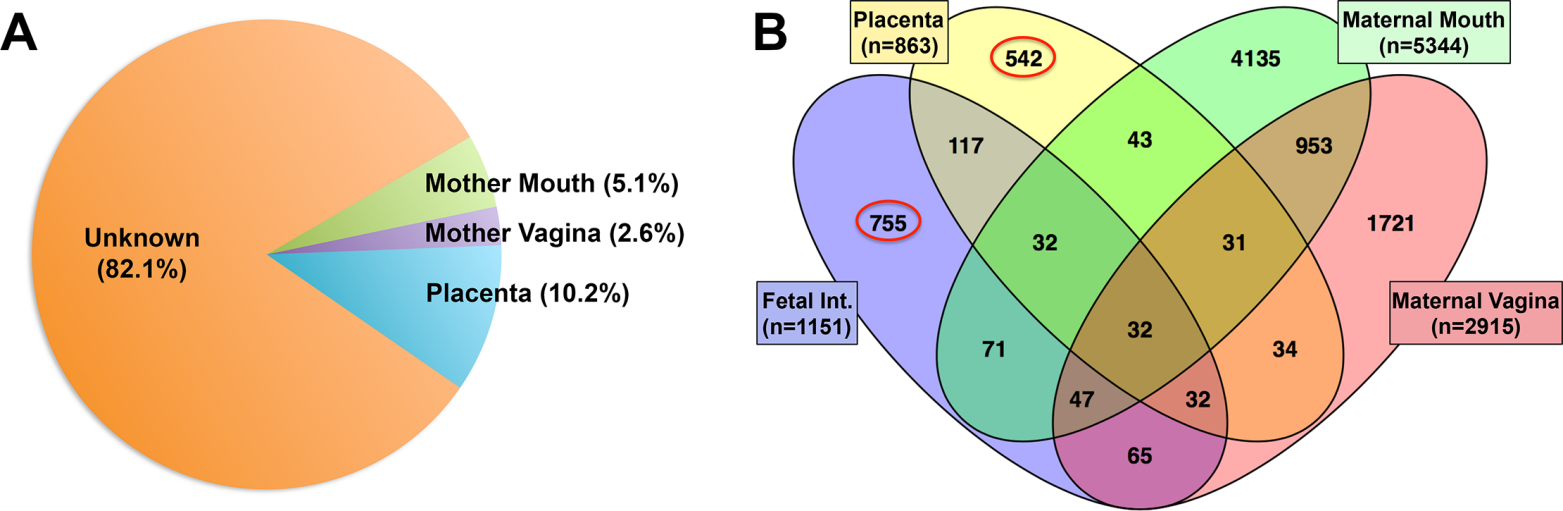
Sources of bacterial DNA-OTUs in the fetal intestine. (A) SourceTracker analysis showing predicted origin of OTUs in placenta, mouth and vagina. (B) Venn diagram showing that 2/3 of the fetal intestine OTUs were not found in placenta, maternal mouth or vagina.

**Fig 4:**
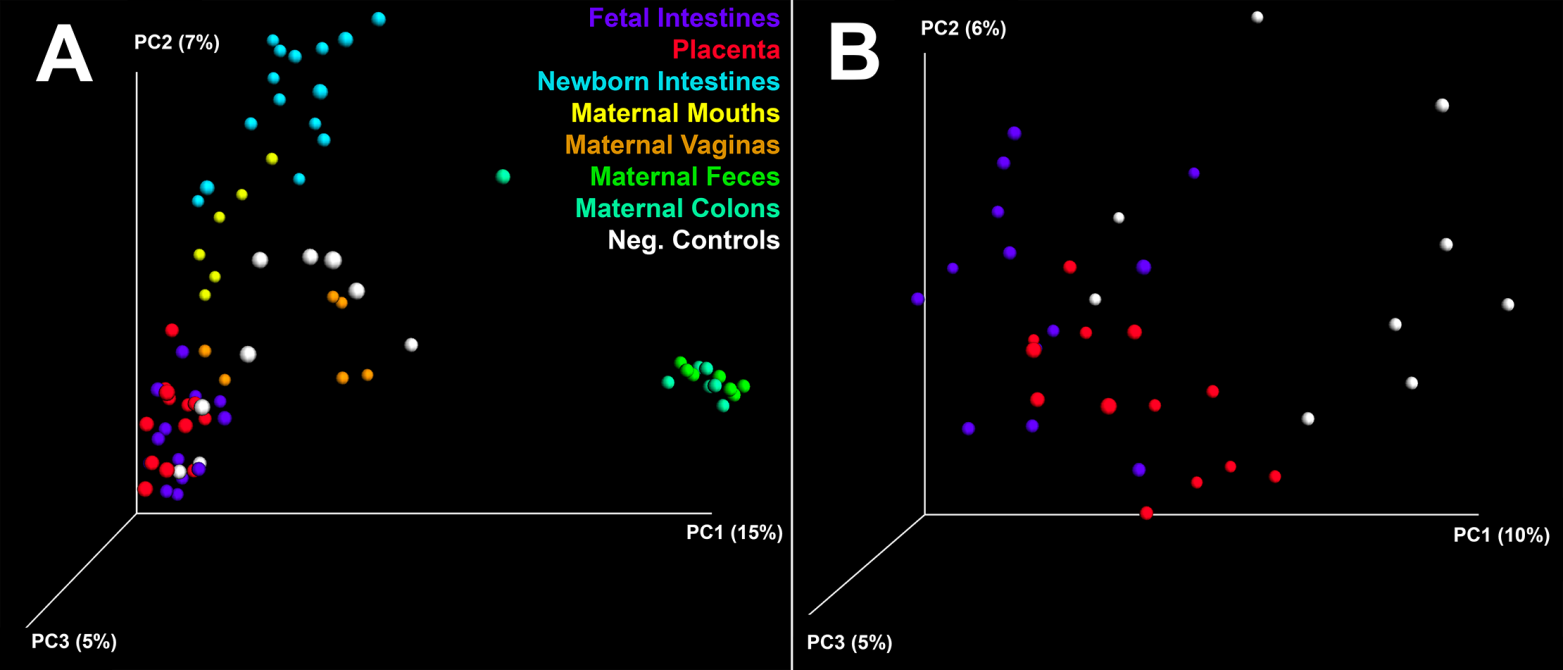
Beta diversity in bacterial DNA from fetal, newborn and maternal samples. (A) Fetal intestine and placenta cluster away from newborn intestines and maternal sites (all PERMANOVA p-values = 0.001). (B) Fetal intestine, placenta and negative controls are significantly different from each other (PERMANOVA p-values = 0.001). Figure generated in QIIME.

SourceTracker analysis revealed the placenta as the most commonly identifiable origin of fetal intestinal bacterial DNA (approximately 10% of fetal intestine OTUs, Fig 3A) while maternal mouth and vagina were the most commonly identified sources for bacterial DNA in the placenta (S3 Fig) and the second most common sources for bacterial DNA in the fetal intestine (Fig 3A). However, many OTUs were found to be common between different sites (Fig 3B). Principal coordinate analysis (PCoA) of unweighted and weighted UniFrac distances, as well as Bray-Curtis similarity, revealed that although OTUs from fetal intestines and placentas segregate apart from all maternal and newborn samples (PERMANOVA p-values 0.001), they also segregate apart from each other (PERMANOVA p-value 0.01) (Fig 4 and S4 Fig) independently of the mother they were sampled from (data not shown). However, linear discriminant analysis effect size (LEfSe) analysis detected no significant differences in genus level taxa level between fetal intestine and placental samples. Litter effect did not account for similarities in bacterial composition between fetal intestines and placentas (S5 Fig).

## Discussion

In this study, we describe the first account of bacterial signatures in mammalian fetal intestinal tissue. The fetal intestinal microbial signature during normal murine pregnancy was unique yet most similar to the placental signature, and after birth, the newborn intestine resembled maternal vaginal and oral microbiomes. Mice tend to lick their newborn pups immediately after birth and we speculate this behavior as a possible rationale for our findings. In addition, the fact that the newborn intestine undergoes a reduction in the diversity of bacterial taxa has been previously reported and is attributable to the selective effect of maternal milk [18].

Low biomass samples, such as the fetal intestine and placenta, require a careful design to minimize contamination [16]. While we cannot completely rule out DNA contamination completely, we exhibited the following cautionary steps: First, we applied stringent experimental practices to reduce contaminants, including UV irradiation of reagents and surfaces, replacement of autoclaved tools between extraction steps (opening of abdominal cavity, extraction of uteri, extraction of fetus), experimental replicates, and maximal surgical barrier precautions for all protocols related to sample preparation. Second, during dissections, we confirmed the absence of environmental contaminants with surface cultures from pregnant dams prepped sterilely and from all fetal samples. Third, we processed negative ‘blank’ controls collected in the same type tubes as tissue samples each time we extracted bacterial DNA from samples and subsequently sequenced these negative controls. Fourth, as is recommended in studies of low bacterial biomass [16], we removed known contaminants detected in sequenced negative controls and reagents from all of our results, including biologically anomalous taxa. Finally, we analyzed sequences independently using two different microbiome analysis software platforms, mothur and QIIME. With these approaches, a diverse fetal microbial DNA signal remained as early as embryonic day 17 (E17) in mice.

Our study has several limitations. First, we did not process collection material, surfaces, or collection tubes in parallel with tissue samples. However, by subtracting control DNAs, we believe we have controlled for contamination issues. Yet, we observe DNA from environmental bacteria such as *Rhodoplanes* (phototrophic bacteria), *Novosphingobium* (water, soil, sediment-dwelling bacteria) and *Stramenopile* chloroplasts (eukaryotic organisms—algae, diatoms) in the fetal intestines (Fig 2). These species may be originating from the diet or drinking water and their DNA might be circulating in the maternal bloodstream. Future studies should include maternal blood samples as controls. Second, we used V4 primers, rather than V1-V3, which may have limited the taxa detected [19]. Other studies with similar objective to ours also targeted the V4 region [1] while others used the V1-V3 sites [10]. A recent metaanalysis in preterm infants found that *Proteobacteria* were more abundant and *Firmicutes* less abundant in studies targeting V3-V5 compared to V1-V3 [20]. However, in this study we compared different body sites with each other and do not emphasize the genera detected. We do not believe that the choice of primers would have altered the principle finding of bacterial DNA detection in the fetal gut.

In this study, we show maternal oral, placental, and fetal bacterial DNA preceding delivery in a murine model. This is consistent with findings in humans, where bacterial DNA has also been reported in placenta, amniotic fluid and meconium [2,9]. Our data also support resemblance between bacterial DNA in the placenta and fetal intestine. The results do not challenge the idea of a sterile fetus, but rather suggest translocation of bacterial DNA from the mother’s microbiome into the fetal intestine. If bacterial DNA is translocated to the fetal intestine through the placenta, its role is unknown. During pregnancy, bacterial components and products are likely disseminated by the maternal vascular supply, as has been shown with regulatory RNAs that cross the placenta [21–23] and may reach the growing fetus. Bacterial DNA transferred from the mother to the fetal gut may be a critical stimulus for normal mucosal immune development [10], but this has not been clearly demonstrated.

Mode of delivery shapes the neonatal microbiome structured by live communities [24], type of feeding (formula versus breast milk), and antibiotic exposure and body site-specific environments largely determine the infant microbiome later in life [21,22], but the maternal microbiome may initiate priming of the fetus immune system, which in turn will modulate the microbiota structure after birth. Further research is needed to elucidate which prenatal interventions could change the fetal microbiome with the goal of improving neonatal outcomes.

## Materials and methods

### Mice

All animal experiments were conducted in accordance with the National Institutes of Health (NIH) animal care standards and were approved by the Vanderbilt University Institutional Animal Care and Use Committee (IACUC). Timed matings of adult C57BL6 mice at 7–10 weeks of age were performed to obtain samples at embryonic day 17 (E17) and post-partum day 1 (P1).

### Maternal sample collection

Following euthanasia with isoflurane, topical calcium hydroxide and potassium thiogycolate was applied to the dams’ abdomen for hair removal prior to disinfection. The abdomen was serially prepped with betadine and isopropyl alcohol. Prepped dams were given to trained laboratory personnel, who resected all maternal samples in a ventilated hood with sterile instruments. All instruments were replaced between mice. In addition, laboratory personnel donned facial masks, surgical caps, sterile surgical gowns, and sterile gloves. Excised gravid uteri were placed inside sterile petri dishes and given to a second trained lab member, who performed all fetal dissections in a separate laminar flow hood as described below. To minimize the possibility of DNA contamination, both hoods were treated with UV light and surfaces wiped with ethanol prior to tissue extractions. Maternal fecal samples from proximal to the colonic lumen were collected in sterile sample vials and immediately snap frozen and stored at -80°C until bacterial DNA extraction.

### Fetal sample collection

To minimize contamination, all fetal dissections were conducted in a sterile, ventilated hood geographically separated from the hood used for maternal dissections. In addition, the dedicated lab person for fetal dissections was never the same person completing maternal dissections. This individual also dressed in sterile surgical garb as described above. New sterile instruments and petri dishes were used to: 1) remove each fetus from the amniotic sac; 2) dissect the placenta from the amniotic tissue; and 3) resect the intestines from the abdominal cavity. For each fetus, placental and intestinal samples were resected, snap frozen, and stored at -80°C.

### Confirming sterile dissection technique

To confirm sterile technique, we performed bacterial cultures of: a) dam’s abdomen before and after sterile surgical preparation; b) oropharynx; c) vagina; and d) peritoneal cavity prior to uterine excision. We collected bacterial cultures from the fetal surface immediately after excision from the amniotic sac and prior to abdominal incision. A bacterial culture was also obtained from the fetal intestinal lumen after resection from the peritoneum. All bacterial cultures were enriched in Luria broth (LB) and incubated at 37°C for 48 hours. Vaginal cultures were also collected in anaerobic transport media and grown for 48 hours in an anaerobic chamber. Bacterial growth was assessed at 24-hour intervals. A positive control, which was a mixture of several non-fastidious bacterial strains, was included with each dissection’s set of bacterial cultures to ensure maintenance of normal bacterial growth conditions. Cultures of placentas, fetal surfaces, and fetal intestines at E17 had no bacterial growth after 48 hours. However, after birth, newborn (P1) intestinal cultures were positive at 24 hours. Cultures of the maternal peritoneum after surgical excision were negative while external maternal abdominal wall cultures were also positive 24 hours prior to, but not after disinfection for surgery. As expected, maternal mouth and vaginal cultures were culture-positive.

### Real-time PCR of fetal and pup intestinal samples

Real-time quantitative (q) PCR amplification was performed in triplicate for all fetal and postnatal pup intestinal samples on an ABI 7900 TaqMan Real Time PCR System (Applied Biosystems, NY) to quantify fetal intestinal bacterial load. We used the conserved eubacterial (EUB) 1114 forward (CGGCAACGAGCGCAACCC) and 1221 reverse (CCATTGTAGCACGTGTGTAGCC) 16S ribosomal primers to detect total bacteria [25]. Reaction mixtures consisted of the 10ng of DNA template, 10μM concentration of each primer, 0.625μL 1X Omni Klentaq (DNA Polymerase technology, cat no. 350), 25mM dNTP (Enzymatics, cat no. N2050L), and 1.25μL EvaGreen (Biotium, CA) in a final reaction volume of 25μL. Cycling conditions were as follows: initial incubation of 95°C for 3 min, denaturing at 95° for 10 sec, then 58 for 30 sec then 72 for 30 sec, for 40 cycles. We used cycle threshold (C_t_) as an indirect indicator for biomass as suggested by others [26]. We normalized C_t_ data by grams of tissue weight and arbitrarily considered a mean C_t >_20 low relative biomass.

### DNA extraction and 16S rRNA gene sequencing

DNA was extracted from fecal and tissue samples using a modified Qiagen protocol that included pretreatment for lysis of Gram-positive bacteria with 20 mg/ml lysozyme in Tris-HCl and EDTA buffer (Qiagen, DNeasy Blood &Tissue Kit, Hilden, Germany) without additional bead beating, as previously reported [14]. The remainder of the DNA extraction protocol proceeded per the manufacturer’s instructions. The V4 region of the 16S rRNA gene from each sample was amplified and sequenced using the Illumina MiSeq Personal Sequencing platforms as described previously [25]. Briefly, 1 μl of DNA template from each sample was added to all reactions and 30 PCR cycles were performed to minimize contaminant amplification. DNA template-free controls (negative controls) were processed concurrently with samples using the same DNA extraction and PCR amplification kits. Negative controls as well as a mixture of bacterial plasmids developed in-house at the Center for Microbial Systems at the University of Michigan for positive PCR control, were sequenced in parallel with study samples. We performed amplifications with barcoded primers, and amplicons from samples and controls were pooled, leaving none excluded.

### Microbiome analysis

The 16S *rRNA* sequence analyses were performed initially with the mothur software package [25,27,28]. For data confirmation and additional illustration, sequences were subsequently analyzed using the QIIME suite of software tools (v1.8) [29]. The sequence reads were used to pick operational taxonomic units (OTUs), with an open-reference OTU picking method based on 97% identity to entries in the Greengenes database (v13_8). Eleven DNA extraction reagent negative controls and 2 PCR reagent negative controls were used. PCR reagent negative controls collected during sampling in same type tubes as study samples were included in the amplification and sequencing procedures to determine background noise and/or contamination, during the processing of the sampling. OTUs from blanks were subtracted from study samples. Of the 11 DNA extractions, 2 had negative controls with extremely high (~2500 each) OTUs and 2 DNA extractions had low efficiency. The negative controls and samples from these DNA extractions were removed from final analysis (analyzed samples and negative controls for each DNA extraction listed in S1 Table and unprocessed sequence information is listed in S2 Table). The negative-control-derived OTUs from the remaining 7 DNA extraction controls were discarded from the OTU table using a filtration script (filter_otus_from_otu_table.py) in QIIME (OTUs found in negative controls are listed in S3 Table). Chimeric sequences were removed using UCHIME [30] prior to analysis. All communities were rarefied to 200 reads per sample because we assumed that low biomass is the cause for low PCR efficiency, resulting in low sequencing yield not surpassing 200 reads per sample in some cases. For comparison of levels of beta diversity between communities, the unweighted/weighted UniFrac distances [31] and Bray-Curtis dissimilarities [32] were calculated and PERMANOVA [33] was used to test significance. We applied linear discriminant analysis effect size (LEfSe) [34] to detect unique biomarkers by determinations of the relative abundances of the members of the bacterial taxonomies.

## Supporting information

S1 Fig Heat maps generated in mothur showing E17 based on operational taxonomic units (OTUs), clustered at 0.03 and classified with RDP classifier. (TIFF)

S2 Fig Heat maps generated in mothur showing P1 based on operational taxonomic units (OTUs), clustered at 0.03 and classified with RDP classifier. (TIFF)

S3 Fig Bacterial OTUs in placental samples. SourceTracker analysis shows maternal mouth and vaginal sources.
(TIF)

S4 Fig Beta diversity in bacterial DNA from fetal, newborn and maternal samples. (A) Weighted UniFrac PCoA generated in QIIME shows fetal intestine (dark blue) and placenta (red) (cluster away from newborn intestines (light blue) and maternal sites (mouth = yellow, vagina = orange, feces = dark green, colon = light green) as well as negative controls (white) and the mock community (gray) (PERMANOVA p-values < 0.05). (B) Bray-Curtis PCoA generated in QIIME shows fetal intestine and placenta cluster away from newborn intestines and maternal sites (PERMANOVA p-values < 0.05).
(TIF)

S5 Fig Litter effect does not account for similarities in bacterial composition between fetal intestines and placentas. NMDS plots generated in mothur comparing the fetal microbiome with those of matched placentas from the same litter for each time point, including E17 (A) and P1 (B). For any given time point, each individual mother-fetus or mother-pup unit is reflected as triangles, circles, or diamonds. Color key for samples is as follows, red: fetal intestines; blue: placenta; green: maternal vagina; orange: maternal mouth; black: maternal stool.
(TIFF)

S1 Table Negative controls and samples processed and analyzed in each DNA extraction. (DOC)

S2 Table Sequencing depth and OTU counts for unfiltered maternal, fetal, placental and newborn samples. (DOC)

S3 Table Number of sequences found in OTUs from negative controls. (DOC)
