# Supplementary figures and images for: Bacterial DNA is present in the fetal intestine and overlaps with that in the placenta in mice
Source: PLoS One. 2018 May 17;13(5):e0197439. doi: 10.1371/journal.pone.0197439 (PMC5957394; doi:10.1371/journal.pone.0197439)

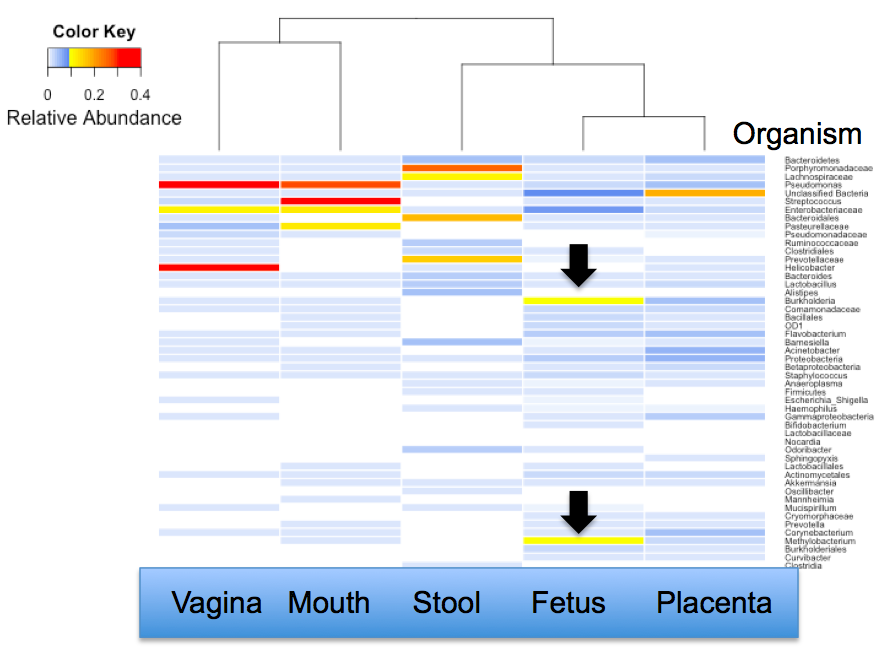

Supplement: S1 Fig — (TIFF) [file pone.0197439.s001.tiff]

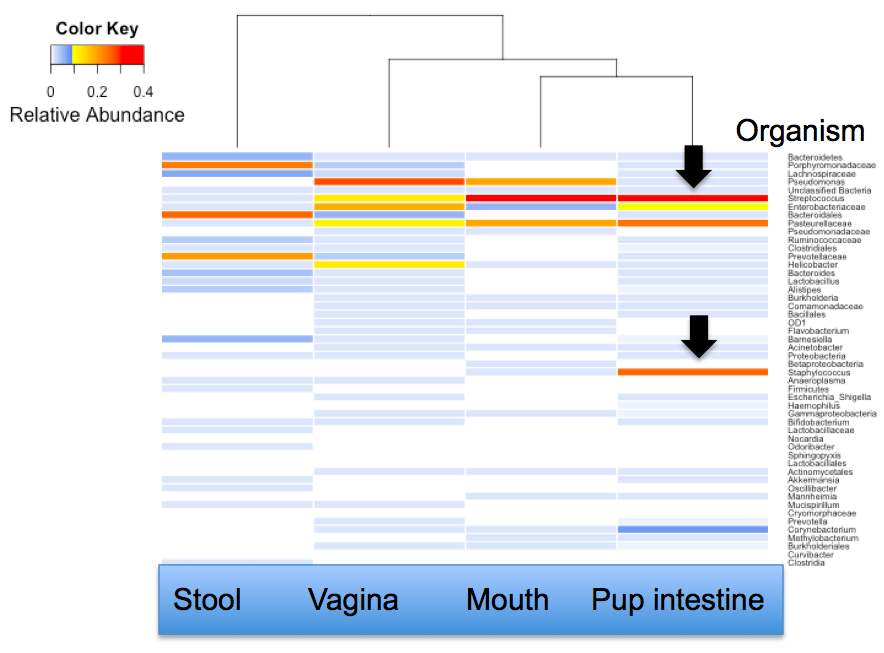

Supplement: S2 Fig — (TIFF) [file pone.0197439.s002.tiff]

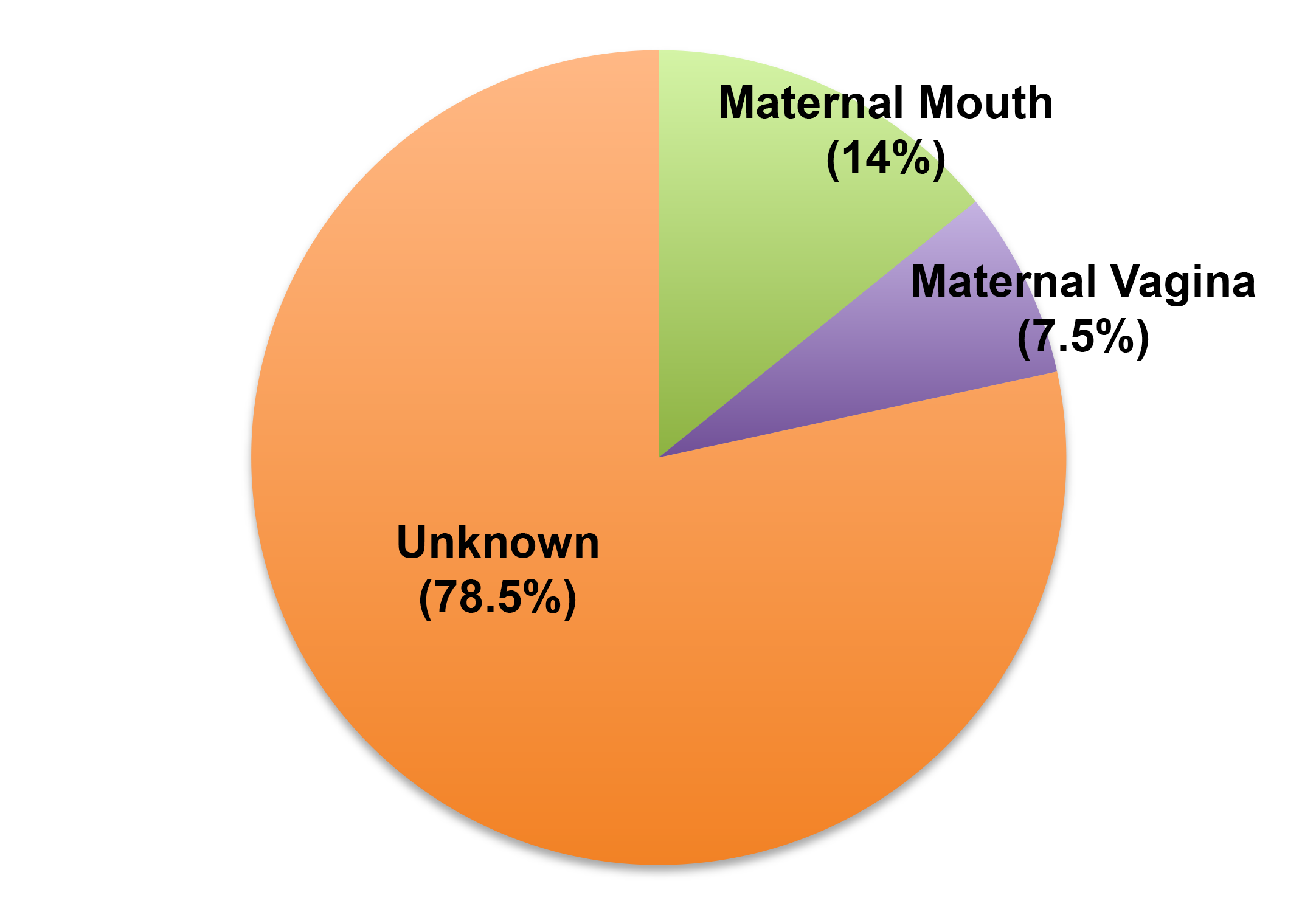

Supplement: S3 Fig — SourceTracker analysis shows maternal mouth and vaginal sources. (TIF) [file pone.0197439.s003.tif]

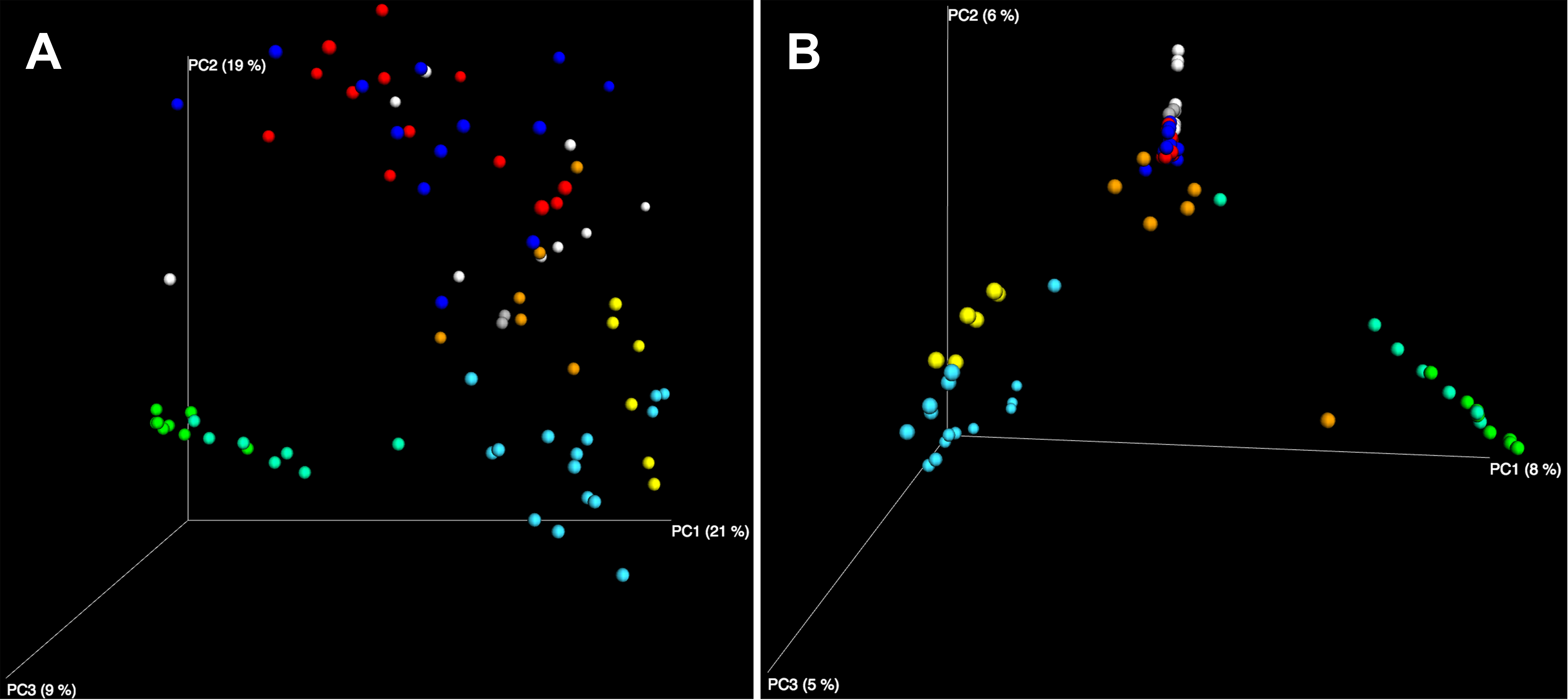

Supplement: S4 Fig — (A) Weighted UniFrac PCoA generated in QIIME shows fetal intestine (dark blue) and placenta (red) (cluster away from newborn intestines (light blue) and maternal sites (mouth = yellow, vagina = orange, feces = dark green, colon = light green) as well as negative controls (white) and the mock community (gray) (PERMANOVA p-values < 0.05). (B) Bray-Curtis PCoA generated in QIIME shows fetal intestine and placenta cluster away from newborn intestines and maternal sites (PERMANOVA p-values < 0.05). (TIF) [file pone.0197439.s004.tif]

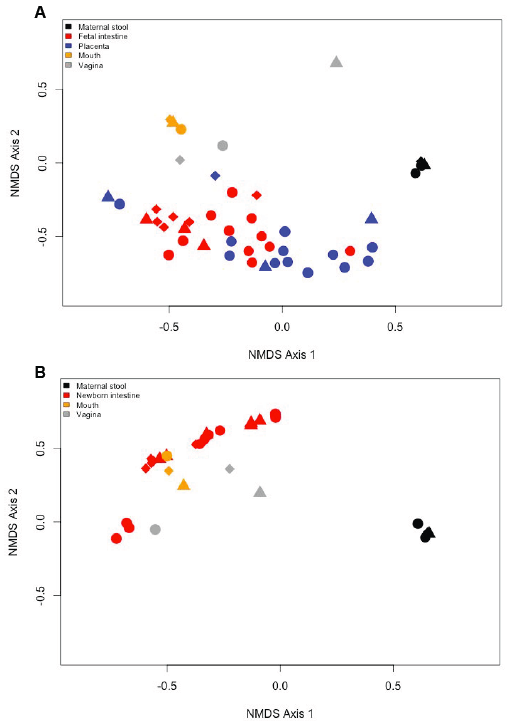

Supplement: S5 Fig — NMDS plots generated in mothur comparing the fetal microbiome with those of matched placentas from the same litter for each time point, including E17 (A) and P1 (B). For any given time point, each individual mother-fetus or mother-pup unit is reflected as triangles, circles, or diamonds. Color key for samples is as follows, red: fetal intestines; blue: placenta; green: maternal vagina; orange: maternal mouth; black: maternal stool. (TIFF) [file pone.0197439.s005.tiff]
