# Supplementary material for: Bacterial DNA is present in the fetal intestine and overlaps with that in the placenta in mice
Source: PLoS One. 2018 May 17;13(5):e0197439. doi: 10.1371/journal.pone.0197439 (PMC5957394; doi:10.1371/journal.pone.0197439)
Supplement: S1 Table — (DOC) [file pone.0197439.s006.doc]

Table S1. Negative controls and samples processed and analyzed in each DNA extraction

| **DNA Extraction** | **Negative Control #** | **Samples** |
| --- | --- | --- |
| 1 | NC3-1 | Fetal Intestine P1 (n=4) |
| 2 | NC3-2 | Fetal Intestine P1 (n=18) |
| 3 | NC4 | Fetal Intestine E17 (n=7), Placenta E17 (n=7) |
| 4 | NC5-1 | Maternal Colon (n=11), Fetal Intestine P1 (n=7) |
| 5 | NC5-2 | Maternal Vagina (n=9), Maternal Mouth (n=9) |
| 6 | NC6-2 | Fetal Intestine E17 (n=7), Placenta E17 (n=7) |
| 7 | NC7-2 | Maternal Feces (n=12) |
