# Supplementary material for: Bacterial DNA is present in the fetal intestine and overlaps with that in the placenta in mice
Source: PLoS One. 2018 May 17;13(5):e0197439. doi: 10.1371/journal.pone.0197439 (PMC5957394; doi:10.1371/journal.pone.0197439)
Supplement: S2 Table — (DOC) [file pone.0197439.s007.doc]

Table S2. Sequencing depth and OTU counts for unfiltered maternal, fetal, placental, and newborn samples

| Sample Type | Maternal | | | | Fetal | | Controls | |
| --- | --- | --- | --- | --- | --- | --- | --- | --- |
|  | Feces | Colon | Oral | Vagina | Placenta | Intestine | PCR Control | Negative Control |
| # of samples | 4 | 4 | 3 | 3 | 23 | 23 | 2 | 6 |
| Total # Seqs to pick OTUs | 201915 | 26965 | 260417 | 239779 | 85816 | 127735 | 6811 | 814341 |
| Mean # of Seqs + St Dev | 50479 + 23882 | 6741 + 1817 | 86806 + 17162 | 79926 + 28591 | 3731+ 2495 | 5554 + 3852 | 3406 + 4523 | 135724 + 116071 |
| # of OTUs represented | 2527 | 748 | 4012 | 1535 | 1829 | 2705 | 326 | 4954 |
| Mean # OTUs  + Std dev | 1042 +251 | 251 +172 | 1872 +209 | 689 +143 | 139 +60 | 227 +89 | 171 +91 | 1181 +753 |
