# Supplementary material for: Bacterial DNA is present in the fetal intestine and overlaps with that in the placenta in mice
Source: PLoS One. 2018 May 17;13(5):e0197439. doi: 10.1371/journal.pone.0197439 (PMC5957394; doi:10.1371/journal.pone.0197439)
Supplement: S3 Table — (DOC) [file pone.0197439.s008.doc]

| OTU ID | Taxonomy | # of Seqs | OTU ID | Taxonomy | # of Seqs |
| --- | --- | --- | --- | --- | --- |
| 923783 | p_Proteobacteria; g_Lysobacter | 169521 | 568118 | p_Bacteroidetes; g_Prevotella | 786 |
| 1064852 | p_Bacteroidetes; g_Flavobacterium | 54024 | 40791 | p_Verrucomicrobia; g_Verrucomicrobium | 767 |
| 1106617 | p_Proteobacteria; f_Comamonadaceae | 48914 | New.ReferenceOTU167 | p_Proteobacteria; o_FAC87 | 761 |
| 1124836 | p_Bacteroidetes; o_Bacteroidales | 44631 | 1048878 | p_Proteobacteria; f_Comamonadaceae | 734 |
| 783719 | p_Proteobacteria; f_Comamonadaceae | 30055 | 816868 | p_Proteobacteria; f_Xanthomonadaceae | 733 |
| 590124 | p_Proteobacteria; g_Serratia | 29963 | 1105280 | p_Proteobacteria; g_Burkholderia | 721 |
| 674655 | p_Proteobacteria; g_Sphingopyxis | 17211 | 817766 | p_Acidobacteria; o_PK29 | 689 |
| New.ReferenceOTU701 | p_Elusimicrobia; o_MVP-88 | 14460 | New.ReferenceOTU47 | p_Proteobacteria; g_Lysobacter | 654 |
| 854050 | p_Firmicutes; g_Bacillus | 12076 | 224900 | p_Proteobacteria; o_Ellin6067 | 651 |
| 666914 | p_Proteobacteria; f_Xanthomonadaceae | 10363 | 273737 | p_Firmicutes; f_Ruminococcaceae | 639 |
| 252003 | p_Proteobacteria; o_Legionellales | 7523 | 873036 | p_Bacteroidetes; g_Fluviicola | 634 |
| 814363 | p_Proteobacteria; g_Ralstonia | 7409 | New.ReferenceOTU400 | p_Bacteroidetes; o_Flavobacteriales | 628 |
| 207635 | p_Firmicutes; g_Paenibacillus | 7148 | 278795 | p_Actinobacteria; g_Rhodococcus | 616 |
| 610486 | p_Proteobacteria; f_Comamonadaceae | 7015 | 1106653 | p_Proteobacteria; f_Sinobacteraceae | 609 |
| 588197 | p_Firmicutes; g_Lactobacillus | 6847 | 357011 | p_Proteobacteria; f_Rhodospirillaceae | 604 |
| New.ReferenceOTU842 | k_Archaea; o_YLA114 | 5683 | 921911 | p_Proteobacteria; o_MIZ46 | 598 |
| New.ReferenceOTU457 | p_Planctomycetes; g_Planctomyces | 4743 | New.ReferenceOTU737 | Unassigned | 593 |
| New.ReferenceOTU257 | p_Cyanobacteria; g_Synechococcus | 4724 | 209628 | p_Verrucomicrobia; f_Opitutaceae | 587 |
| 591521 | p_Actinobacteria; g_Mycobacterium | 4322 | New.ReferenceOTU648 | p_Proteobacteria; g_Lysobacter | 586 |
| 1087471 | p_Bacteroidetes; o_Sphingobacteriales | 4147 | New.ReferenceOTU57 | Unassigned | 548 |
| New.ReferenceOTU82 | p_Cyanobacteria; g_Synechococcus | 4117 | 4366724 | p_Proteobacteria; g_C39 | 545 |
| 243500 | p_Proteobacteria; f_Burkholderiaceae | 4068 | 529793 | p_Actinobacteria; f_EB1017 | 531 |
| 582558 | p_Proteobacteria; g_Sulfuricurvum | 3866 | 220892 | p_Verrucomicrobia; o_Pedosphaerales | 510 |
| 832674 | p_Proteobacteria; g_Janthinobacterium | 3832 | 267046 | p_Bacteroidetes; f_S24-7 | 498 |
| 240252 | p_Proteobacteria; g_Acidocella | 3473 | New.CleanUp.ReferenceOTU463834 | p_Fibrobacteres | 493 |
| 1013213 | p_Proteobacteria; f_Comamonadaceae | 3162 | New.CleanUp.ReferenceOTU548211 | Unassigned | 486 |
| 564487 | p_Bacteroidetes; g_Pedobacter | 2953 | 148003 | p_Proteobacteria; g_Serratia | 476 |
| 2554 | p_Bacteroidetes; o_Saprospirales | 2817 | 284266 | p_OD1; c_ABY1 | 470 |
| New.ReferenceOTU805 | Unassigned | 2758 | New.ReferenceOTU343 | p_Proteobacteria; f_Rhodospirillaceae | 453 |
| New.ReferenceOTU110 | p_Bacteroidetes; f_Chitinophagaceae | 2727 | New.CleanUp.ReferenceOTU415437 | Unassigned | 453 |
| 274011 | p_Thermi; g_Truepera | 2652 | 544177 | p_Proteobacteria; f_Pseudomonadaceae | 434 |
| 1032692 | p_Bacteroidetes; o_Sphingobacteriales | 2627 | 580571 | p_Proteobacteria; f_Oxalobacteraceae | 431 |
| 270707 | p_Bacteroidetes; f_S24-7 | 2612 | 552685 | p_Actinobacteria; o_Actinomycetales | 421 |
| New.ReferenceOTU679 | p_OD1; c_ZB2 | 2598 | 564411 | p_Proteobacteria; f_Oxalobacteraceae | 420 |
| 200762 | p_GN02; c_BD1-5 | 2309 | New.CleanUp.ReferenceOTU489812 | Unassigned | 406 |
| New.ReferenceOTU863 | Unassigned | 2296 | New.CleanUp.ReferenceOTU270261 | p_Planctomycetes; o_WD2101 | 401 |
| 777498 | p_Proteobacteria; g_Methylotenera | 2165 | 572138 | p_Bacteroidetes; f_Chitinophagaceae | 400 |
| New.ReferenceOTU268 | p_Proteobacteria; c_Deltaproteobacteria | 2113 | 1034564 | p_Bacteroidetes; g_Chryseobacterium | 390 |
| 2760486 | p_Elusimicrobia; o_Elusimicrobiales | 1997 | 1086621 | p_Proteobacteria; g_Burkholderia | 379 |
| 363400 | p_Firmicutes; f_Lachnospiraceae | 1997 | 1106324 | p_Proteobacteria; g_Limnohabitans | 364 |
| New.ReferenceOTU836 | p_Proteobacteria; g_Methylobacterium | 1993 | 151096 | p_Bacteroidetes; o_Bacteroidales | 357 |
| 337724 | p_Bacteroidetes; f_S24-7 | 1690 | New.ReferenceOTU28 | Unassigned | 349 |
| New.ReferenceOTU724 | p_Planctomycetes; o_agg27 | 1674 | New.CleanUp.ReferenceOTU95869 | p_Chlamydiae; f_Parachlamydiaceae | 349 |
| 1055322 | p_Bacteroidetes; g_Flavobacterium | 1665 | 274749 | p_Bacteroidetes; f_S24-7 | 347 |
| 1107461 | p_Firmicutes; o_Clostridiales | 1652 | 4344472 | p_Acidobacteria; f_Holophagaceae | 346 |
| New.ReferenceOTU818 | p_Firmicutes; g_vadinHB04 | 1651 | 650048 | p_OD1; c_ZB2 | 344 |
| 1034645 | p_Bacteroidetes; g_Flavobacterium | 1588 | New.ReferenceOTU379 | p_Firmicutes; g_Paenibacillus | 329 |
| 539244 | p_Bacteroidetes; g_CF231 | 1542 | New.ReferenceOTU622 | p_Proteobacteria; g_Lysobacter | 325 |
| 348038 | p_Bacteroidetes; f_S24-7 | 1489 | New.CleanUp.ReferenceOTU294040 | Unassigned | 318 |
| 189721 | p_Bacteroidetes; g_Prevotella | 1467 | 430194 | p_Bacteroidetes; f_S24-7 | 316 |
| 547148 | p_Gemmatimonadetes; c_Gemm-1 | 1459 | New.CleanUp.ReferenceOTU81832 | p_Proteobacteria; o_Spirobacillales | 311 |
| 113767 | p_TM7; c_SC3 | 1454 | 571406 | p_Firmicutes; g_Eubacterium | 302 |
| New.ReferenceOTU725 | p_Proteobacteria; f_Xanthomonadaceae | 1414 | 81488 | p_Proteobacteria; f_Xanthomonadaceae | 296 |
| New.ReferenceOTU686 | p_Proteobacteria; g_Bdellovibrio | 1399 | New.CleanUp.ReferenceOTU643943 | p_Cyanobacteria; g_Synechococcus | 295 |
| 759916 | p_Proteobacteria; g_Ralstonia | 1350 | New.CleanUp.ReferenceOTU573917 | Unassigned | 291 |
| 754778 | p_Proteobacteria; g_Erwinia | 1338 | 1110763 | p_Proteobacteria; f_Enterobacteriaceae | 285 |
| 672144 | p_Proteobacteria; g_Delftia | 1329 | New.ReferenceOTU228 | p_Proteobacteria; g_Lysobacter | 282 |
| New.ReferenceOTU383 | p_Proteobacteria; g_Lysobacter | 1277 | New.ReferenceOTU230 | p_Bacteroidetes; o_Saprospirales | 282 |
| New.CleanUp.ReferenceOTU480502 | p_Bacteroidetes; o_Saprospirales | 1274 | 518474 | p_Proteobacteria; f_Legionellaceae | 280 |
| New.ReferenceOTU851 | p_Bacteroidetes; g_Flavobacterium | 1252 | New.CleanUp.ReferenceOTU155690 | p_OD1; c_ZB2 | 279 |
| New.ReferenceOTU693 | p_Firmicutes; g_Caloramator | 1145 | New.CleanUp.ReferenceOTU80404 | p_OD1; c_ZB2 | 277 |
| 278073 | p_OD1; c_SM2F11 | 1118 | 276575 | p_Firmicutes; f_Lachnospiraceae | 276 |
| 312378 | p_Elusimicrobia; o_FAC88 | 1081 | 1105814 | p_Proteobacteria; f_Bradyrhizobiaceae | 275 |
| 699027 | p_Bacteroidetes; g_Flavobacterium | 1003 | 282328 | p_TM7; c_SC3 | 272 |
| New.CleanUp.ReferenceOTU159891 | Unassigned | 995 | 856536 | p_Proteobacteria; o_Myxococcales | 270 |
| 1108282 | p_Firmicutes; f_Bacillaceae | 992 | New.CleanUp.ReferenceOTU244377 | Unassigned | 269 |
| 1111294 | p_Proteobacteria; f_Enterobacteriaceae | 969 | New.CleanUp.ReferenceOTU446211 | p_Bacteroidetes; g_Fluviicola | 264 |
| 1039594 | p_Proteobacteria; f_Comamonadaceae | 950 | 837283 | p_Proteobacteria; g_Serratia | 263 |
| 3240079 | p_Cyanobacteria; o_SM1D11 | 923 | 279019 | p_Firmicutes; o_Clostridiales | 257 |
| 447141 | p_Bacteroidetes; f_S24-7 | 895 | New.ReferenceOTU731 | p_Proteobacteria; g_Lysobacter | 247 |
| 157434 | p_Bacteroidetes; o_Bacteroidales | 883 | New.CleanUp.ReferenceOTU504098 | p_Proteobacteria; o_Spirobacillales | 247 |
| 583388 | p_Bacteroidetes; g_Flavobacterium | 849 | 677064 | p_Proteobacteria; f_Oxalobacteraceae | 244 |
| New.ReferenceOTU56 | p_Proteobacteria; f_Methylocystaceae | 839 | 1116384 | p_Proteobacteria; f_Comamonadaceae | 231 |
| New.ReferenceOTU524 | p_Firmicutes; f_Acidaminobacteraceae | 800 | 573326 | p_Bacteroidetes; g_Chryseobacterium | 228 |

Table S3. Number of sequences found in OTUs from negative controls

| OTU ID | Taxonomy | # of Seqs |
| --- | --- | --- |
| 707290 | p_Proteobacteria; f_Xanthomonadaceae | 223 |
| New.ReferenceOTU850 | p_TM7; c_TM7-1 | 223 |
| 819037 | p_Proteobacteria; g_Limnohabitans | 221 |
| 1105696 | p_Firmicutes; g_Clostridium | 220 |
| 4379780 | p_Proteobacteria; o_Myxococcales | 218 |
| New.CleanUp.ReferenceOTU622658 | p_Proteobacteria; f_Xanthomonadaceae | 217 |
| 810955 | p_Bacteroidetes; g_Chryseobacterium | 216 |
| New.CleanUp.ReferenceOTU469760 | p_Bacteroidetes; f_Saprospiraceae | 216 |
| New.ReferenceOTU488 | p_Cyanobacteria; g_Synechococcus | 215 |
| New.ReferenceOTU264 | p_Proteobacteria; f_Comamonadaceae | 214 |
| 814832 | p_Bacteroidetes; g_Flavobacterium | 211 |
| 728991 | p_Bacteroidetes; g_Bacteroides | 211 |
| New.CleanUp.ReferenceOTU536931 | p_Proteobacteria; o_MIZ46 | 208 |
| New.ReferenceOTU341 | p_Proteobacteria; g_Lysobacter | 203 |
| 971457 | p_Bacteroidetes; g_Flavobacterium | 203 |
| 584177 | p_Proteobacteria; g_Delftia | 199 |
| New.CleanUp.ReferenceOTU419638 | p_Proteobacteria; f_Xanthomonadaceae | 193 |
| New.CleanUp.ReferenceOTU26536 | p_Planctomycetes; f_Pirellulaceae | 193 |
| 798634 | p_Proteobacteria; f_Comamonadaceae | 191 |
| 356568 | p_Proteobacteria; g_Methylotenera | 188 |
| New.ReferenceOTU541 | p_Proteobacteria; g_Lysobacter | 184 |
| New.ReferenceOTU366 | p_Proteobacteria; f_Xanthomonadaceae | 182 |
| 222534 | p_Proteobacteria; f_Xanthomonadaceae | 174 |
| New.ReferenceOTU435 | p_Proteobacteria; f_Burkholderiaceae | 173 |
| 982582 | p_Proteobacteria; g_Lysobacter | 173 |
| New.ReferenceOTU651 | p_Proteobacteria; g_Lysobacter | 172 |
| New.ReferenceOTU709 | p_Bacteroidetes; o_Bacteroidales | 166 |
| New.CleanUp.ReferenceOTU31864 | p_Proteobacteria; o_BD7-3 | 166 |
| 1068936 | p_Actinobacteria; o_Acidimicrobiales | 164 |
| New.CleanUp.ReferenceOTU529204 | Unassigned | 163 |
| 591499 | p_Proteobacteria; f_Oxalobacteraceae | 161 |
| New.CleanUp.ReferenceOTU62769 | p_Cyanobacteria; g_Synechococcus | 161 |
| 2233608 | p_Bacteroidetes; f_S24-7 | 160 |
| 756083 | p_Proteobacteria; g_Arcobacter | 158 |
| 461206 | p_Bacteroidetes; g_Flavobacterium | 157 |
| 781203 | p_Bacteroidetes; g_Sediminibacterium | 154 |
| New.CleanUp.ReferenceOTU107007 | Unassigned | 151 |
| New.ReferenceOTU180 | p_Firmicutes; f_Bacillaceae | 147 |
| New.CleanUp.ReferenceOTU380013 | p_Proteobacteria; g_Methylobacterium | 146 |
| New.ReferenceOTU669 | p_Proteobacteria; g_Lysobacter | 137 |
| 370357 | p_Firmicutes; o_Clostridiales | 137 |
| 105352 | p_Actinobacteria; f_Microbacteriaceae | 137 |
| New.ReferenceOTU430 | p_Proteobacteria; c_Deltaproteobacteria | 136 |
| 2304693 | p_Proteobacteria; g_Serratia | 135 |
| 338142 | p_Proteobacteria; g_Ralstonia | 133 |
| 827051 | p_Bacteroidetes; g_Sediminibacterium | 132 |
| New.CleanUp.ReferenceOTU82093 | p_Proteobacteria; o_MIZ46 | 127 |
| 1110008 | p_Firmicutes; f_Ruminococcaceae | 124 |
| 261350 | p_Bacteroidetes; f_S24-7 | 124 |
| New.CleanUp.ReferenceOTU604464 | Unassigned | 124 |
| New.CleanUp.ReferenceOTU119970 | Unassigned | 122 |
| New.ReferenceOTU566 | p_Elusimicrobia; c_Endomicrobia | 121 |
| New.CleanUp.ReferenceOTU476654 | p_Cyanobacteria; g_Synechococcus | 121 |
| 141856 | p_Bacteroidetes; f_Cryomorphaceae | 120 |
| New.CleanUp.ReferenceOTU419845 | p_TM6; c_SJA-4 | 120 |
| 124309 | p_Proteobacteria; g_Serratia | 118 |
| New.CleanUp.ReferenceOTU177798 | p_Cyanobacteria; g_Synechococcus | 118 |
| 842284 | p_Proteobacteria; f_Xanthomonadaceae | 117 |
| 624227 | p_Actinobacteria; o_Actinomycetales | 115 |
| 633252 | p_Proteobacteria; g_Pseudomonas | 114 |
| New.CleanUp.ReferenceOTU42821 | p_Acidobacteria; f_Holophagaceae | 114 |
| New.ReferenceOTU763 | p_Cyanobacteria; g_Synechococcus | 113 |
| New.CleanUp.ReferenceOTU444721 | p_Cyanobacteria; g_Synechococcus | 113 |
| New.ReferenceOTU245 | p_Proteobacteria; f_Xanthomonadaceae | 110 |
| 348395 | p_Proteobacteria; f_Comamonadaceae | 110 |
| 714231 | p_Proteobacteria; f_Xanthomonadaceae | 106 |
| 560336 | p_Bacteroidetes; g_Bacteroides | 106 |
| New.CleanUp.ReferenceOTU124900 | p_Proteobacteria; o_PHOS-HD29 | 105 |
| New.CleanUp.ReferenceOTU606755 | p_Cyanobacteria; o_Euglenozoa | 104 |
| 4416113 | p_Proteobacteria; g_Serratia | 102 |
| OTUs with 50-100 sequences |  | 86 |
| OTUs with 1-10 sequences |  | 2926 |
| Singleton OTUs |  | 1773 |
